# A Novel Combined Nomogram Model for Predicting the Pathological Complete Response to Neoadjuvant Chemotherapy in Invasive Breast Carcinoma of No Specific Type: Real-World Study

**DOI:** 10.3389/fonc.2022.916526

**Published:** 2022-06-06

**Authors:** Xuelin Zhu, Jing Shen, Huanlei Zhang, Xiulin Wang, Huihui Zhang, Jing Yu, Qing Zhang, Dongdong Song, Liping Guo, Dianlong Zhang, Ruiping Zhu, Jianlin Wu

**Affiliations:** ^1^Graduate School, Tianjin Medical University, Tianjin, China; ^2^Department of Radiology, Affiliated Zhongshan Hospital of Dalian University, Dalian, China; ^3^Department of Ultrasound, Qingzhou People's Hospital, Weifang, China; ^4^Department of Radiology, Yidu Central Hospital of Weifang, Weifang, China; ^5^School of Biomedical Engineering, Faculty of Electronic Information and Electrical Engineering, Dalian University of Technology, Dalian, China; ^6^Department of Ultrasound, Affiliated Zhongshan Hospital of Dalian University, Dalian, China; ^7^Department of Breast and Thyroid Surgery, Affiliated Zhongshan Hospital of Dalian University, Dalian, China; ^8^Department of Pathology, Affiliated Zhongshan Hospital of Dalian University, Dalian, China

**Keywords:** multiparametric MRI, radiomics, neoadjuvant chemotherapy, invasive breast carcinoma of no specific type, pathologic complete response

## Abstract

**Objective:**

To explore the value of a predictive model combining the multiparametric magnetic resonance imaging (mpMRI) radiomics score (RAD-score), clinicopathologic features, and morphologic features for the pathological complete response (pCR) to neoadjuvant chemotherapy (NAC) in invasive breast carcinoma of no specific type (IBC-NST).

**Methods:**

We enrolled, retrospectively and consecutively, 206 women with IBC-NST who underwent surgery after NAC and obtained pathological results from August 2018 to October 2021. Four RAD-scores were constructed for predicting the pCR based on fat-suppression T2-weighted imaging (FS-T2WI), diffusion-weighted imaging (DWI), contrast-enhanced T1-weighted imaging (T1WI+C) and their combination, which was called mpMRI. The best RAD-score was combined with clinicopathologic and morphologic features to establish a nomogram model through binary logistic regression. The predictive performance of the nomogram was evaluated using the area under receiver operator characteristic (ROC) curve (AUC) and calibration curve. The clinical net benefit of the model was evaluated using decision curve analysis (DCA).

**Results:**

The mpMRI RAD-score had the highest diagnostic performance, with AUC of 0.848 among the four RAD-scores. T stage, human epidermal growth factor receptor-2 (HER2) status, RAD-score, and roundness were independent factors for predicting the pCR (*P* < 0.05 for all). The combined nomogram model based on these factors achieved AUCs of 0.930 and 0.895 in the training cohort and validation cohort, respectively, higher than other models (*P* < 0.05 for all). The calibration curve showed that the predicted probabilities of the nomogram were in good agreement with the actual probabilities, and DCA indicated that it provided more net benefit than the treat-none or treat-all scheme by decision curve analysis in both training and validation datasets.

**Conclusion:**

The combined nomogram model based on the mpMRI RAD-score combined with clinicopathologic and morphologic features may improve the predictive performance for the pCR of NAC in patients with IBC-NST.

## Introduction

For females, breast cancer (BC) is the leading cause of cancer in 157 countries and the leading cause of death in 119 countries (1). Neoadjuvant chemotherapy (NAC) for BC is a systemic therapy using a cytotoxic drug administered before definitive surgical treatment (2). As a personalized precision treatment approach, the purpose of NAC is to: (i) reduce tumor stage; (ii) treat potential metastatic lesions in a timely manner; (iii) observe the sensitivity of tumors to chemotherapy regimens to provide a basis for the selection of subsequent treatment regimens (3, 4). However, about 20% of BC patients are not sensitive to NAC, and a few even experience disease progression during treatment (5). Meanwhile, chemotherapeutic drugs can also lead to adverse effects (e.g., bone-marrow suppression, liver and kidney impairment, heart failure) in some patients (6, 7). Therefore, evaluating BC patients before chemotherapy and predicting if they will benefit from NAC are crucial. pCR is the most widely used surrogate endpoint for NAC efficacy assessment, and patients achieving pCR may have higher disease-free survival and overall survival (8). Hence, early prediction of pCR may help to improve personalized treatment plans and even avoid surgery in the future.

Conventional imaging medicine obtains the morphological characteristics of tumor phenotypes through visual assessment by radiologists, which can provide an overall image of the tumor phenotype and its environment. These morphological features (e.g., shape, border, lobing) observed originally by the naked eye are dichotomous variables based on two-dimensional (2D) sections. Some scholars have suggested that they can be characterized by quantitative data using mathematical formulae in which “roundness” can indicate the shape of the lesion, “concavity” reflects the irregularity of the lesion border, and “curvature” describes the morphologic changes of breast-tumor lesions (9). They are all based on quantitative measurements, so refining dichotomous variables into digital variables would be advantageous compared with using conventional morphologic qualitative features assessed by the naked eye.

Dynamic contrast enhanced magnetic resonance imaging (DCE-MRI) is one of the most sensitive methods for early prediction of pCR, which can reflect changes in tissue pathophysiology before morphological changes (10). In addition, T2 weighted imaging (T2WI), diffusion weighted imaging (DWI) and other sequences have also been used in the prediction of NAC for BC (11, 12).

Multiparametric magnetic resonance imaging (mpMRI) involves combined application of several MRI imaging sequences. mpMRI can be employed to quantify the evolution of cancer development at multiple levels and dimensions, and provide specific quantitative information about tumor characteristics. Compared with single-sequence MRI models, mpMRI improves the diagnostic accuracy of BC and the evaluation and prediction performance of NAC efficacy (13).

Radiomics uses automated algorithms to convert the image data of the region of interest in medical images into high-dimensional spatial data, and then extracts the key information that really works from a large amount of information through a variety of statistical analysis and data mining methods. Then, the obtained information is applied to support systems for clinical decision-making to aid disease characterization, tumor staging, efficacy assessment, and prognosis prediction (14). Compared with conventional imaging, radiomics fully reflects the most essential characteristics of the underlying medical images. In recent years, radiomics based on mpMRI has developed rapidly and become a “hotspot” for basic research and clinical applications, and has made great progress in BC and other research fields (15–19).

Invasive breast carcinoma of no specific type (IBC-NST) is the most common type of pathologic staging for BC. It accounts for about 70–80% of cases, and is characterized by low differentiation and a poor prognosis (20, 21). Few studies have been conducted to predict the pathologic complete response (pCR) of NAC based on mpMRI radiomics for people with IBC-NST.

We aimed to establish and validate a nomogram model based on mpMRI radiomics, clinicopathologic features, and morphologic features for early prediction of pCR in IBC-NST.

## Materials and Methods

### Ethical Approval of the Study Protocol

The protocol for this retrospective study was approved (2018068) by the Ethics Committee of Zhongshan Hospital Affiliated to Dalian University (Dalian, China). The requirement for written informed consent from study participants was waived.

### Patients

The inclusion criteria were: (i) female BC patients over 18 years old who came to our hospital for treatment; (ii) MRI was performed and immunohistochemical results were obtained by ultrasound-guided needle biopsy before NAC; (iii) surgery was performed after NAC, and pCR was confirmed by postoperative pathologic examination. The exclusion criteria were: (i) a specific type of invasive breast cancer; (ii) MRI findings were obtained >1 week before NAC; (iii) not receiving a standardized and complete NAC regimen or other related treatment previously; (iv) lesions were combined with other sites of primary cancer; (v) lesions were combined with distant metastases; (vi) the quality of the MR image was insufficient to obtain measurements; (vii) the correlation between the tumor and assessment of the pathologic response in MR images was uncertain; (viii) incomplete clinical or pathologic data.

**Figure 1:**
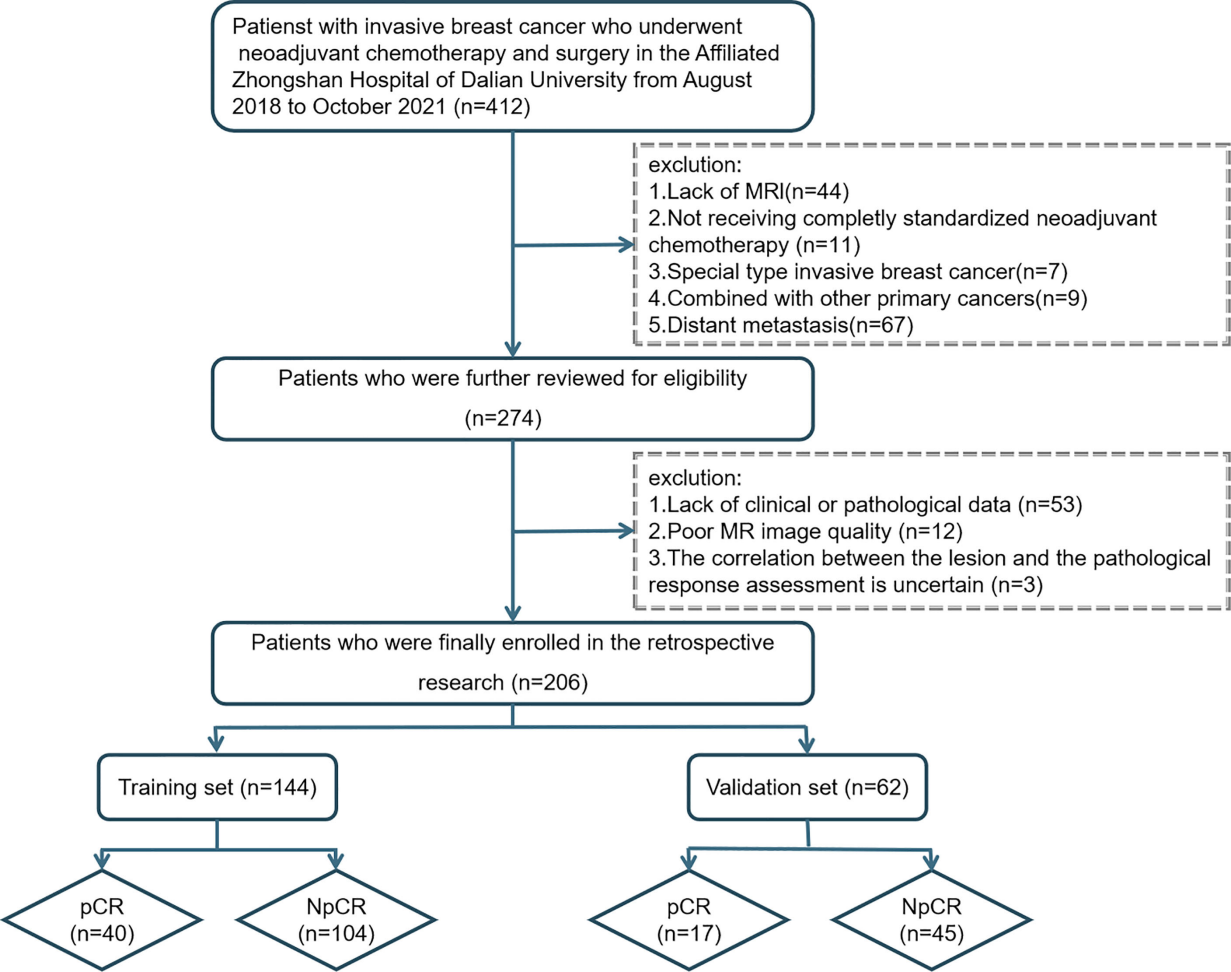
Flowchart revealing the study population based on exclusion criteria.

Patients suffering from BC who underwent NAC and a surgical procedure at Zhongshan Hospital from August 2018 to October 2021 were included retrospectively and consecutively. Patients were divided into a pCR group and non-pathologic complete response (NpCR) group according to whether pCR was achieved after NAC. Enrolled patients were assigned randomly to a training cohort and a validation cohort at a ratio of 7:3. The training cohort is used for model establishment, and the validation cohort is used for model performance verification. A flowchart showing the study population is presented as **Figure 1**.

Baseline clinical data were obtained from medical records. The age, body mass index (BMI), menopause status, fibrinogen, T stage, and N status of patients before NAC were collected. Treatment regimens and treatment cycles followed the National Comprehensive Cancer Network guideline (22). All BC patients completed ≥4 cycles of NAC with: (i) paclitaxel-based chemotherapy (4.4%, 9/206); (ii) anthracycline-based chemotherapy (21.8%, 45/206); (iii) anthracycline-based chemotherapy combined with paclitaxel-based chemotherapy (73.8%, 152/206). Human epidermal growth factor receptor 2 (HER2)-positive patients also received trastuzumab or/and pertuzumab (35.4%, 73/206). All patients underwent surgery at our hospital within 2 weeks of completing a full cycle of NAC. Analyses of pathologic histologic sections and diagnosis were undertaken by two pathologists with 12 years and 10 years of experience in the diagnosis of breast disease, respectively, who were blinded to the study protocol. Pre-NAC status of the estrogen receptor (ER), progesterone receptor (PR), HER2, and Ki-67 expression were obtained from immunohistochemical analyses of the puncture specimen (for assessment criteria see **Supplemental Data 1**). pCR was confirmed by postoperative pathological examination. The “pCR” was defined as an absence of residual invasive carcinoma in the specimen (residual ductal carcinoma *in situ* can be present), ipsilateral anterior sentinel lymph node or no lymph node infiltration in lymph nodes removed during axillary dissection (23).

### MRI Protocol

All MR images were acquired on a 3.0-T Magnetom Verio superconducting MRI scanner equipped with a 16-channel breast-specific coil (Siemens, Hamburg, Germany) within one week prior to NAC. Imaging sequences were fat-suppression T2-weighted imaging (FS-T2WI), diffusion-weighted imaging (DWI, contrast-enhanced T1-weighted imaging (T1WI+C). Scan parameters are shown in **Supplemental Data 2**.

### Extraction and Selection of Morphologic Features

**Figure 2:**
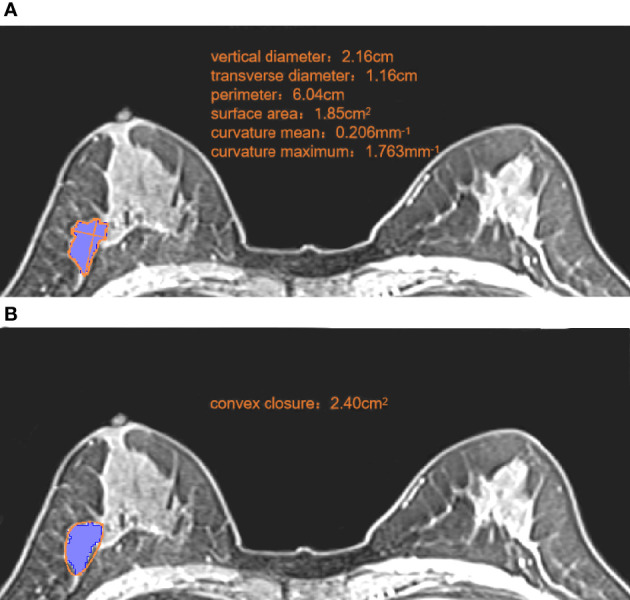
Measurement of morphologic parameters. **(A)** We measured the vertical diameter and transverse diameter at the largest cross-section. We drew along the edge of the lesion, and obtained the perimeter, surface area, curvature mean, and curvature maximum. Roundness **=**4π ×1.85/6.04^2^ = 0.64 **(B)** We made a small convex polygon along the line connecting the edge of the lesion to obtain the convex closure area. Concavity= (2.40-1.85)/2.40 = 0.23.

MR images were evaluated independently by two radiologists, A and B, with 11 years and 14 years of experience of diagnosing BC using MRI, respectively. Measurements were made using a double-blind method (neither radiologist was aware of clinicopathologic findings or the other radiologist’s interpretation of images). Morphologic features were measured using 3D Slicer (version 4.11, www.slicer.org/). The maximum cross-section of the T1WI+C sequence was used for measurement, and the following values measured under the “Markups” module: vertical diameter, transverse diameter, perimeter, surface area, convex closure area, curvature maximum, and curvature mean (**Figure 2**). Roundness was calculated using the formula: 4π × surface area/perimeter². Concavity was calculated indirectly from the measured convex closure area: (convex closure area – surface area)/convex closure area (24). Thirty cases were randomly selected from the enrolled population before assignment, and the repeatability of feature extraction was assessed using intra-observer and inter-observer intraclass correlation coefficients (ICCs). Each parameter was measured twice, and the mean value calculated: this was used as the final measurement. Multicollinearity was used to reduce morphologic features, and parameters with a variance inflation factor (VIF) <10 were selected for subsequent analyses.

### Tumor Segmentation and Radiomics Feature Extraction

The region of interest (ROI) was delineated manually *via* 3D Slicer (version 4.11, www.slicer.org/) on each slice of the FS-T2WI, DWI (b-value of 800s/mm²), and T1+C (second period after contrast agent injection) image sets (DICOM format) of all cases. On each slice of the images, necrotic, air, and calcified regions were excluded.

Two radiologists (A and B) were responsible for the evaluation of tumor segmentation. Inter- and intra-observer reproducibility of radiomic feature extraction were initially analyzed with the data of 30 randomly selected patients from each sequence in a double-blinded fashion by these 2 radiologists. The ICCs were used to evaluate the agreement of radiomics features.

Prior to feature extraction, all images were resampled to a common voxel spacing of 1mm × 1mm × 1mm by using the Resize method, to resample the images into an isotropic dataset to allow comparison between image data from different sequences.

The “Radiomics” module in the 3D Slicer (version 4.11, www.slicer.org/) was used to extract 1223 radiomics features from each sequence of FS-T2WI, DWI, and T1WI+C. One hundred and seven radiomic features were extracted from the original images, including 18 first-order features, 14 shape features, and 75 texture features derived from the gray level co-occurrence matrix (GLCM, 24), gray level dependence matrix(GLDM, 14), gray level run length matrix (GLRLM, 16), gray level size zone matrix (GLSZM, 16) and neighbourhood gray-tone difference matrix (NGTDM, 5). With a Laplacian of Gaussian filter, 372 features with four sigma levels (0.5, 1, 1.5, 2) were obtained. A total of 744 features were obtained from 8 derived images by wavelet transform.

### Selection of Radiomics Features and Construction of a “RAD-Score”

All extracted features were normalized by z-score in the training cohort before selecting radiomic features. First, features with ICC >0.75 within the training cohort were retained. Second, *t*-tests or *u*-tests were carried out on the retained features, and we targeted those with discriminatory ability (*P* < 0.05) for further analyses. Third, we applied the least absolute shrinkage and selection operator (LASSO) regression for selecting the key radiomics features with nonzero coefficients, and a 10-fold cross-validation with a maximum area under the receiver operator characteristic (ROC) curve (AUC) criterion was conducted to determine an optimal regulation weight (lambada). After the steps stated above, the remaining features were subjected to RAD-score construction. The above-mentioned RAD-scores were constructed for FS-T2WI, DWI, T1WI+C sequences and mpMRI, respectively. The sequence with the best diagnostic efficacy RAD-score was selected for entry into subsequent analyses. The RAD-score of each patient is linear combinations of selected features weighted by their coefficients, which are mathematically represented as follows:


$$RAD-score=\sum_{i=1}^{n}C_{i}\times X_{i}+b$$

where b is the intercept, X_i_ is the ith selected feature, and C_i_ is the coefficient of the ith selected feature. Feature selection and RAD-score construction were performed using the “glmnet” package of R software (version 4.1.4, www.r-project.org/).

### Development of Prediction Models

Prediction models were developed using univariate and multifactorial logistic regression based on clinicopathologic, morphologic, and radiomics features. Clinicopathologic features included age, menopausal status, fibrinogen level, BMI, T stage, N stage, receptor status (ER, PR, HER2), and Ki-67 expression. Features with P < 0.05 after univariate analysis were included in the multifactorial analysis. Next, we development 3 models based on features of different categories. Model A was established based on clinicopathologic features. Model B was established based on clinicopathologic and radiomics features. Model C was established based on clinicopathologic, radiomics, and morphologic features.

### Comparison of the Performance of Prediction Models

**Figure 3:**
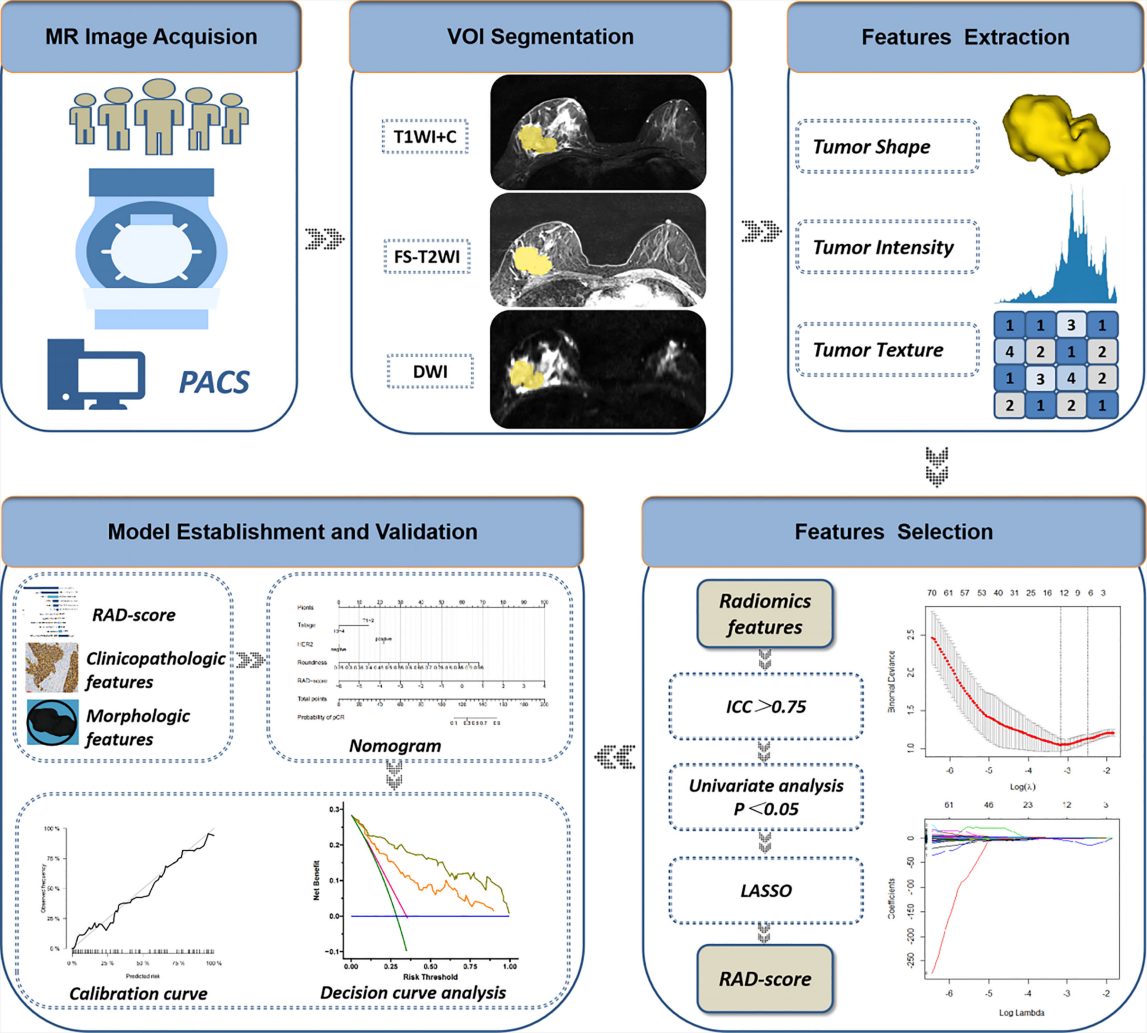
Flowchart of extraction of radiomics features, model establishment and performance evaluation. ICC, intraclass correlation coefficient; LASSO, least absolute shrinkage and selection operator; RAD-score, radiomics score.

Accuracy, specificity, sensitivity, and the area under receiver operator characteristic curve (AUC) were used to estimate the predictive performance of the three models in the training cohort and validation cohort. The model with the best performance was presented as a nomogram. Then, a calibration curve was used to evaluate the consistency between the estimated probability and actual probability of the pCR. Decision curve analysis (DCA) was used to assess the clinical usefulness by estimating the net benefit within threshold probabilities. A flowchart of extraction of radiomics features and model establishment is shown as **Figure 3**.

### Statistical Analyses

Differences between pCR and NpCR groups were analyzed using the *t*-test, *u*-test, or chi-square test. Statistical analyses were undertaken using R software (version 4.1.4, www.r-project.org/). Within R software, the packages “performance” and “see” were employed for multicollinearity analyses. “glmnet” were used for feature selection and RAD-score construction, and “pROC”, “rms”, “Hmisc” and “ggDCA” were employed for model construction and performance evaluation. P < 0.05 was considered significant.

## RESULTS

### Patient Characteristics

A total of 206 BC patients formed the study cohort. They had a median age of 52 (range, 42–60) years. Among them, 57 (27.7%) were in the pCR group and 149 (72.3%) were in the NpCR group. There was no significant difference in age, menopausal status, fibrinogen level, body mass index, or lymph-node metastasis between pCR and NpCR groups (P > 0.05 for all). T stage, ER status, PR status, HER2 status, and Ki-67 expression were significantly different between the groups (P < 0.05 for all) (**Table 1**).

**Table 1:** Demographic and clinical characteristics of the study cohort.

Variable	All patients (n = 206)	NpCR group (n = 149)	pCR group (n = 57)	*P-*value
Age, median	52 (42, 60)	52 (42, 59)	51 (42, 61)	0.889
Menopausal status, n (%)				0.640
Pre	103 (50.0)	76 (51.0)	27 (47.4)	
Post	103 (50.0)	73 (49.0)	30 (52.6)	
BMI, median	24.65 (22.28, 26.64)	24.65 (22.6, 26.29)	24.58 (20.7, 26.81)	0.181
FIB, median	2.69 (2.42, 3.07)	2.69 (2.46, 3.1)	2.65 (2.37, 3.02)	0.387
T Stage, n (%)				0.019
T1–2	114 (55.3)	65 (49.2)	49 (66.2)	
T3–4	92 (44.7)	67 (50.8)	25 (33.8)	
N Status, n (%)				0.631
Negative	33 (16.0)	25 (16.8)	8 (14.0)	
Positive	173 (84.0)	124 (83.2)	49 (86.0)	
ER Status, n (%)				<0.001
Negative	79 (38.4)	39 (26.2)	40 (70.0)	
Positive	127 (61.6)	110 (73.8)	17 (29.8)	
PR Status, n (%)				<0.001
Negative	95 (46.1)	52 (34.9)	43 (75.4)	
Positive	111 (53.9)	97 (65.1)	14 (24.6)	
HER2 Status, n (%)				<0.001
Negative	133 (64.6)	111 (74.5)	22 (38.6)	
Positive	73 (35.4)	38 (25.5)	35 (61.4)	
Ki-67 Status, n (%)				0.010
Low expression	42 (20.4)	37 (24.8)	5 (8.8)	
High expression	164 (79.6)	112 (75.2)	52 (91.2)	
Cohort, n (%)				0.695
Training cohort	144 (69.9)	103 (69.1)	41 (71.9)	
Validation cohort	62 (30.1)	46 (30.9)	16 (28.1)	

pCR, pathologic complete response; NpCR, non-pathologic complete response; BMI, body mass index; FIB, fibrinogen; ER, estrogen receptor; PR, progesterone receptor; HER2, human epidermal growth factor receptor 2.

All patients were divided randomly into a training cohort (144) and a validation cohort (62) at a ratio of 7∶3. In the training cohort, 41 cases had a pCR and 103 had a NpCR. In the validation cohort, 16 patients had a pCR and 46 had a NpCR. There was no significant differences pCR ratio between the training cohort and validation cohort (P > 0.05) (**Table 1**).

### Morphologic Features

**Table S1** shows that all eight morphologic variables have ICCs >0.9 (i.e., showed good agreement). Multicollinearity analysis (**Figure S1**) meant that the final remaining two variables (roundness and concavity) entered the next step of analysis.

### Selection of Radiomics Features and Establishment of the RAD-Score

**Figure 4:**
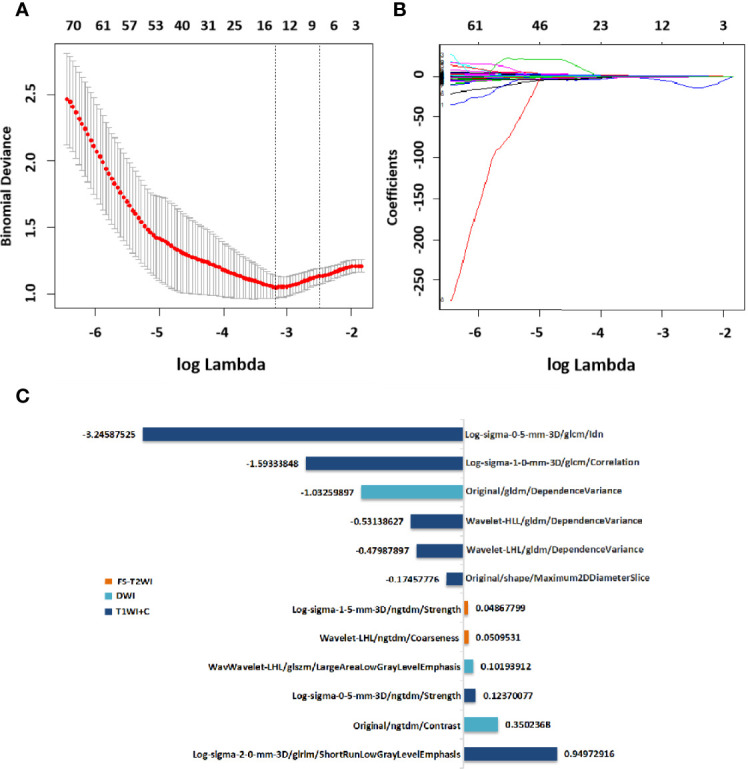
Selection of radiomics features *via* LASSO algorithm to establish a RAD-score. **(A)** Tuning parameter selection by 10-fold cross-validation with minimum criteria. Mean square error (y-axis) was plotted as a function of log(lambda) (x-axis). **(B)** LASSO coefficient profiles for the whole features. **(C)**12 radiomics features corresponding to the selected optimal values for establishment of a RAD-score. LASSO, least absolute shrinkage and selection operator; RAD-score, radiomics score.

After screening, the mpMRI sequence has 12 remaining features, including 1 shape feature, 2 original texture features, 5 Gaussian filter transformation features and 4 wavelet features. Texture features, Gaussian filter features and wavelet features are based mainly on the GLCM, GLDM, NGTDM, and GLSZM.The selection process operated by LASSO is represented in **Figure 4**. Reduction of feature dimensionality and the display of each sequence feature are shown in **Figure S2** and **Table S2**, respectively. The RAD-score was calculated as follows:

RAD-score= (T1WI+C/Log-sigma-0-5-mm-3D/ngtdm/Strength×0.12370077) -(T1WI+C/Original/shape/Maximum2DDiameterSlice×0.17457776) -(T1WI+C/Log-sigma-1-0mm-3D/glcm/Correlation×1.59333848) -(T1WI+C/Log-sigma-0-5-mm-3D/glcm/Idn×3.24587525) +(T1WI+C/Log-sigma-2-0-mm-3D/glrlm/ShortRunLowGrayLevel Emphasis×0.94972916) -(T1WI+C/Wavelet-LHL/gldm/DependenceVariance×0.479 87897) -(T1WI+C/Wavelet-HLL/gldm/DependenceVariance×0.53138627) -(DWI/Original/gldm/DependenceVariance×1.03259897) +(DWI/Original/ngtdm/Contrast×0.35 02368) +(DWI/Wavelet-LHL/glszm/LargeAreaLowGrayLevelEmphasis×0.1019391 2) +(FS-T2WI/Log-sigma-1-5-mm-3D/ngtdm/Strength×0.04867799) +(FS-T2WI/Wavelet-LHL/ngtdm/Coarseness×0.05095310) +4.59154672

The features shown by mpMRI had the highest diagnostic performance in the training cohort (AUC=0.848) and validation cohort (AUC =0.742), followed by T1WI+C sequence, with an AUC of 0.799 in the training cohort and 0.741 in validation cohort. For the FS-T2WI sequence, the AUC of training cohort was 0.737 and the AUC of validation cohort was 0.635, while DWI sequence yielded an AUC of 0.750 in training cohort and 0.626 in validation cohort. (**Figure S3**).

### Construction of Predictive Models

**Figure 5:**
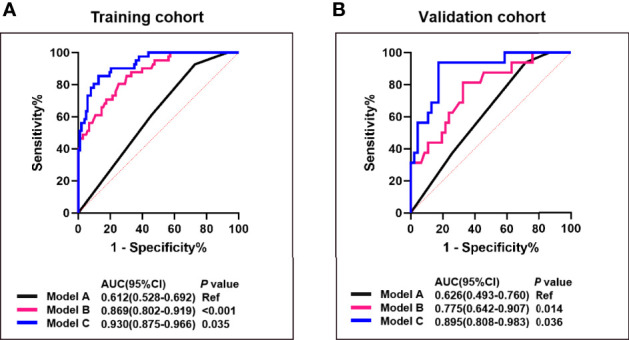
Comparison of the predictive performance between model A, B and (C) ROC curves and AUCs for predicting pCR of model A (blue curve), model B (pink curve), and model C (black curve) in the training **(A)** and the validation cohort **(B)**. Model A, T stage + HER2 status; Model B, model A + RAD-score; Model C, model B + roundness.

**Figure 6:**
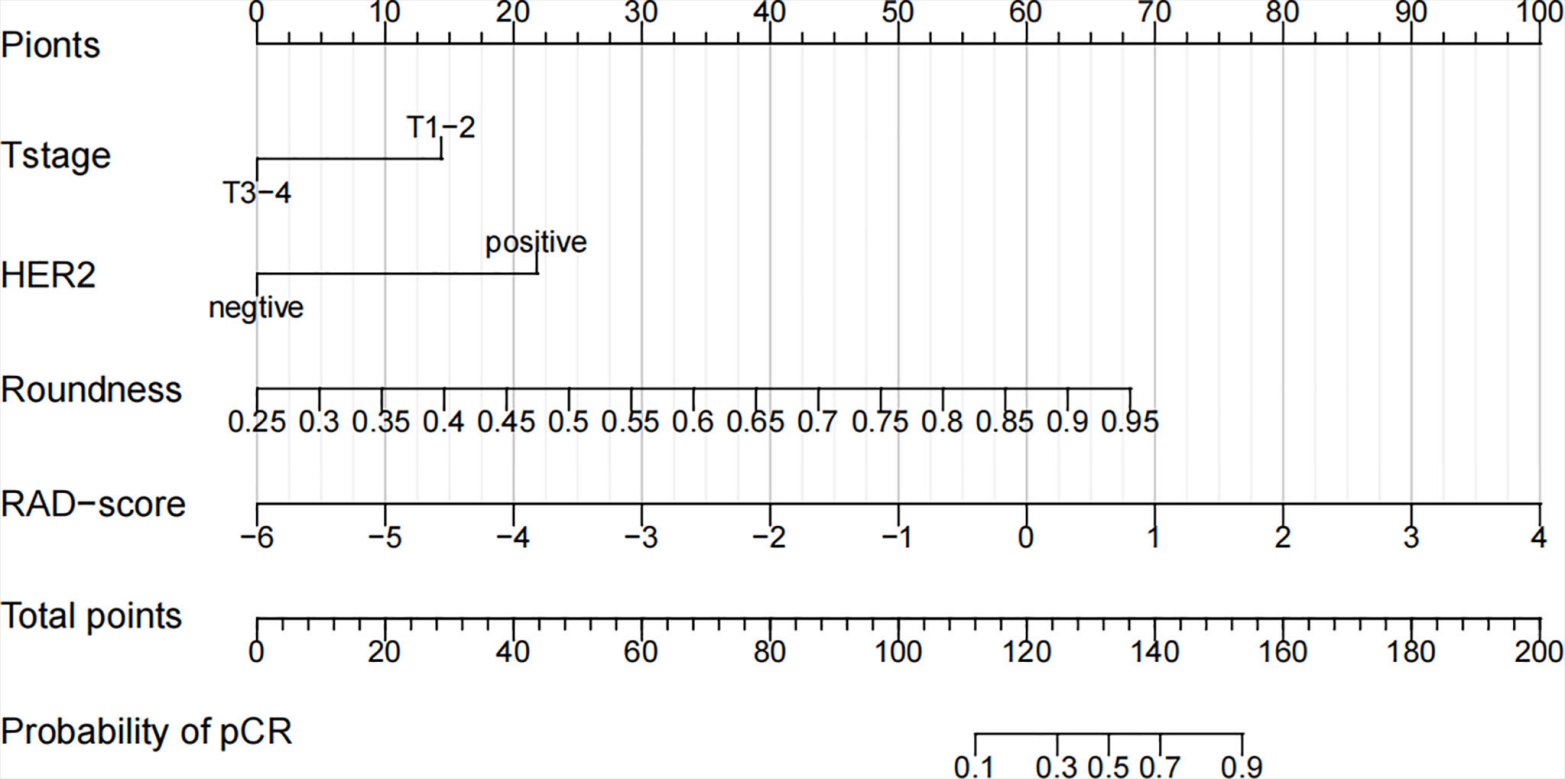
A nomogram for the prediction of the pathological complete response of neoadjuvant chemotherapy in invasive breast carcinoma of no specific type.

In the training cohort, univariate logistic regression analysis showed that T stage, HER2 status, roundness, and RAD-score were potential predictors (P < 0.05) which were associated with pCR status. Then, the variables stated above were included in the multivariate logistic regression analysis for the construction of the 3 models: model A (T stage + HER2 status), model B (Model A + RAD-score), and model C (Model B + roundness) (**Table 2**). The AUC of model A in the training cohort was 0.612 (95% CI, 0.528-0.692) and in the validation cohort was 0.626 (95% CI, 0.493-0.760). Model B yielded an AUC of 0.869 (95% CI, 0.802-0.919) and an AUC of 0.775 (95% CI, 0.642-0.907). Compared to the other 2 models, model C exhibited the highest discrimination performance in the training cohort (AUC, 0.930; 95% CI, 0.875-0.966) and validation cohort (AUC, 0.895; 95% CI, 0.808-0.983) (**Figure 5**). Accuracy, sensitivity, specificity, and AUC of each model are shown in **Table 3**. Model C is shown in a nomogram (**Figure 6**).

**Table 2:** Multivariate logistic analysis.

Variable	Model A		Model B		Model C	
	OR (95%CI)	*P-*value	OR (95%CI)	*P*-value	OR (95%CI)	*P*-value
T stage	4.47 (2.33–9.59)	0.035	2.266 (1.132–5.397)	0.044	2.354 (1.023–5.236)	0.036
HER2 status	2.56 (1.83–7.89)	0.012	3.713 (1.677–8.291)	0.009	1.947 (1.320–4.321)	0.024
RAD-score			5.057 (2.031–11.851)	0.026	3.911 (1.591–9.642)	0.003
Roundness					7.554 (3.2151–7.664)	<0.001

HER2, human epidermal growth factor receptor. RAD-score, radiomics score; OR, odds ratio; CI, confidence interval.

**Table 3:** Performance of prediction models in the training cohort and validation cohort.

Model	Cohort	AUC (95%CI)	P*	SE	SP	ACC
**Model A**	Training cohort	0.612 (0.528-0.692)	Ref	0.927	0.272	0.458
	Validation cohort	0.626 (0.493-0.760)	Ref	0.626	0.261	0.355
**Model B**	Training cohort	0.869 (0.802-0.919)	<0.001	0.854	0.699	0.743
	Validation cohort	0.775 (0.642-0.907)	0.014	0.813	0.674	0.710
**Model C**	Training cohort	0.930 (0.875-0.966)	0.035	0.854	0.874	0.868
	Validation cohort	0.895 (0.808-0.983)	0.036	0.938	0.826	0.855

Model A, T stage + HER2; Model B, Model A + RAD-score; Model C, Model B + roundness; AUC, the area under receiver operator characteristic curve; CI, confidence interval; P*, Delong test; SE, sensitivity; SP, specificity; ACC, accuracy.

**Figure 7:**
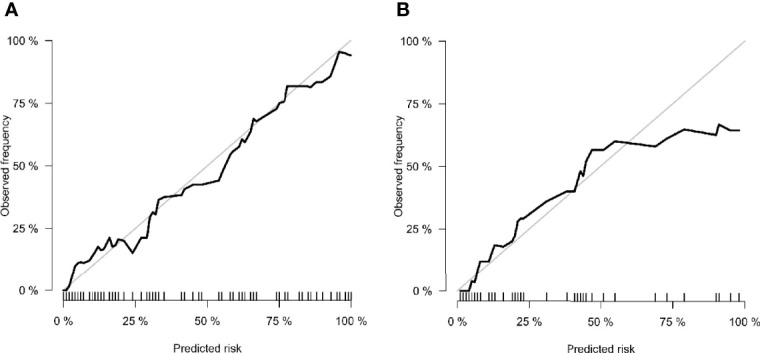
Calibration curves of **(A)** training cohort and **(B)** validation cohort. The x-axis is the nomogram-predicted probability. The y-axis is the observed probability. The closer fit of the diagonal curved line to the ideal straight line indicates the predictive accuracy of the nomogram from the best model.

**Figure 8:**
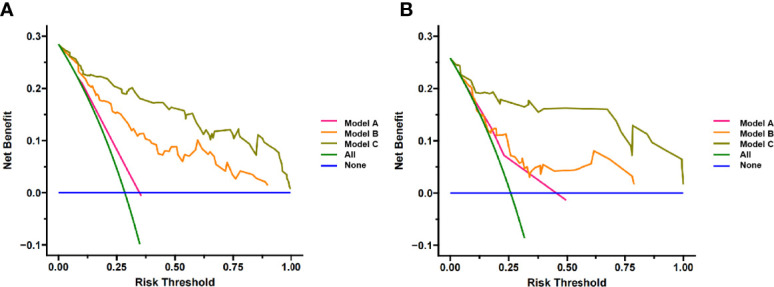
Decision Curve analysis for three models in **(A)** training cohort and **(B)** validation cohort. The y-axis measures the net benefit. The pink line represents model A, the orange line represents model B and the brown line represents model C. The green line represents the assumption that all patients gained substantial benefit after NAC. The horizontal blue line represents the assumption that no patients gained substantial benefit after NAC.

The calibration curve of the nomogram showed that the predicted results were in good agreement with the actual results **(** **Figure 7**). The result of the DCA indicated that the prediction of pCR using model C could give more net benefit than by treating none or all patients in both training and validation datasets (**Figure 8**).

## Discussion

Using an IBC-NST population, we developed a nomogram model based on the RAD-score of mpMRI combined with clinicopathologic and morphologic features. This combined model had high value for predicting the pCR prior to NAC. The performance of this model was better than that of the clinical model, or the model combining clinical features with radiomics features.

We established RAD-score models for FS-T2WI, DWI, T1WI+C, and mpMRI to predict the pCR before NAC, respectively. The mpMRI RAD-score model had the highest diagnostic performance in the training cohort (AUC=0.848) and validation cohort (AUC =0.742). Among the other three single-sequence models, the T1WI+C model performed the best with AUCs of 0.799 and 0.741 in the training and validation cohorts, respectively. This indicates that T1WI+C is one of the most sensitive methods for predicting pCR in the single MRI sequence, which is also consistent with many previous studies (25, 26). Whereas in studies comparing mpMRI with single sequence, Chen et al. (27) evaluated 91 patients and found that in the pCR prediction models, the radiomics signature of mpMRI exhibited higher predictive power (AUC = 0.848) compared to DCE(AUC=0.750) and ADC(AUC=0.785). Bian et al. (28) also found that the mpMRI model had the highest predictive power for the pCR in a study of 152 patients, with AUC of 0.91 and 0.93 in the training and validation cohort. Using a larger cohort, we demonstrated that combined application of different imaging sequences was superior to that using a single sequence. Under a logistic algorithm model, *t*-test, *U*-test, and LASSO regression were employed to select the optimal number of features. Within the range of standard deviation of the highest AUC value, 12 features were selected for the mpMRI RAD-score (T1WI+C7, DWI 3, FS-T2WI 2). These features included one shape feature, four wavelet features, and seven texture features. Among them, the only shape feature was the maximum 2D diameter slice of the T1WI+C sequence, which reflected the tumor diameter. The smaller the tumor, the easier the pCR could be achieved.

Texture features, Gaussian filter features and wavelet features are based mainly on the GLCM, GLDM, NGDTM, and GLSZM. The GLCM provides comprehensive information about the direction, adjacent interval, and variation range of the gray level of the image. The GLCM is the basis for analyzing the local patterns of the image and their arrangement rules, and is used to describe the texture distribution and characteristics within the tumor. The NGTDM describes the visual characteristics of texture based on a voxel and its neighborhood. The GLSZM can aid characterization of texture consistency, a periodic texture, or speckle texture (29). These are high-order features and cannot be identified by the naked eye. However, they can capture information on the spatial heterogeneity of intratumoral cells and tumor perfusion, thereby making them sensitive for treatment evaluation (30). Therefore, the RAD-score could serve as a non-invasive imaging marker for pCR prediction.

We found that the T stage and HER2 status, as clinicopathologic features, were independent influencing factors of the pCR. The T stage represents the diameter of tumor tissue and the extent of tumor invasion. Briete et al. (31) postulated clinical tumor stage to be the most important predictor of a pCR in BC patients after NAC. We further confirmed that a lower T stage (T1–2 and T3-4) is an important independent predictor of a higher prevalence of a pCR.

HER2 is a prognostic indicator for monitoring of clinical treatment and an important target for selection of tumor-targeting drugs. Several studies (5, 32, 33) have suggested that HER2-positivity can lead to a higher prevalence of the pCR: we reached the same conclusion. Also, we found roundness to be an independent predictor of the pCR after NAC. The roundness of a tumor represents the shape of the lesion. The value of roundness is between 0 and 1. The closer the value is to 1, the closer is the shape to a circle (34). Based on the most intuitive impression by radiologists, roundness quantifies shape features and is accepted readily by clinicians. Few studies have shown the relationship between roundness and the pCR. However, Zhang et al. (35) concluded, in a retrospective analysis of 120 BC patients, that the roundness observed in triple-negative BC patients was higher than that observed in non-triple-negative BC patients whereas, in general, triple-negative BC responded better to NAC than other molecular subtypes. Their data supported our findings indirectly. In a multicenter study by Liu et al. (36), the radiomics signature of mpMRI achieved an AUC of 0.79 (the highest of the four radiomics signatures), whereas a prediction model combining the RAD-score and clinicopathologic characteristics before NAC had higher predictive value for the pCR (AUC = 0.86). A study by Zhang et al. (37) obtained similar results (AUC = 0.84). On the basis of the radiomics features of mpMRI combined with clinicopathologic features, we also added roundness (a quantitative morphologic feature) and narrowed the study cohort to IBC-NST patients: the AUC of the combined prediction model reached 0.930. The clinical value must be validated further, but addition of quantitative morphologic factors aids improvement of the predictive power of the model.

Our study had four main limitations. First, this was a retrospective, single-center study, and a selection bias was inevitable. Second, the heterogeneity of molecular subtypes included in our study led to the use of different chemotherapy regimens, this scenario is in accordance with clinical practice, but may lead simultaneously to an imbalance in the pathologic response of NAC. Third, we carried out comprehensive internal verification, which demonstrated the reliability and repeatability of the constructed model to a certain extent, and reduced the risk of a confounding bias. However, the conclusions of our study are based only on a particular population. Verification in multicenter studies is needed to improve the universality of our model. Fourth, the sequences used in the mpMRI radiomics were FS-T2WI, DWI, and T1WI+C. Whether combination with other sequences or even other imaging modalities (e.g., ultrasound, computed tomography, positron emission tomography) can help improve the prediction performance must be explored further. Meanwhile, the comparison of multiple classifiers and the application of deep learning will also become our future research directions.

## Conclusions

We developed a combined nomogram model based on mpMRI radiomics, clinicopathologic features, and morphologic features for early prediction of pCR to NAC in IBC-NST. Compared with models based on clinicopathologic features alone or combining clinicopathologic and radiomic features, this model has higher predictive performance and is expected to provide more references for making decisions on clinical treatment in the future.

## Data Availability Statement

The original contributions presented in the study are included in the article/**Supplementary Material**. Further inquiries can be directed to the corresponding author.

## Ethics Statement

This study was reviewed and approved by the Ethics Committee of Affiliated Zhongshan Hospital of Dalian University, and patient informed consent was waived.

## Author Contributions

XZ, JS, HLZ, and JW conceived the study. XZ, JS, and HHZ collected the data. HLZ, XW, and DS analyzed the data. XZ wrote the manuscript. JW, JY, QZ, and LG provided study supervision. DZ and RZ conducted the histological assessments. All authors contributed to the article and approved the submitted version.

## Funding

This study is supported by Scientific Research Project Plan of Weifang Health Commission (WFWSJK−2021−071).

## Conflict of Interest

The authors declare that the research was conducted in the absence of any commercial or financial relationships that could be construed as a potential conflict of interest.

## Publisher’s Note

All claims expressed in this article are solely those of the authors and do not necessarily represent those of their affiliated organizations, or those of the publisher, the editors and the reviewers. Any product that may be evaluated in this article, or claim that may be made by its manufacturer, is not guaranteed or endorsed by the publisher.
