# Supplementary material for: A Novel Combined Nomogram Model for Predicting the Pathological Complete Response to Neoadjuvant Chemotherapy in Invasive Breast Carcinoma of No Specific Type: Real-World Study
Source: Front Oncol. 2022 Jun 6;12:916526. doi: 10.3389/fonc.2022.916526 (PMC9207207; doi:10.3389/fonc.2022.916526)
Supplement: Supplementary file 1 [file DataSheet_1.docx]

Supplementary Material

# Supplementary Data

**Supplemental data 1**

The status of the estrogen receptor (ER), progesterone receptor (PR), human epidermal growth factor receptor-2 ((HER2), and Ki-67 before neoadjuvant chemotherapy for breast cancer was obtained from immunohistochemical (IHC) analyses of puncture specimens. ER and PR tumor cell nuclei stained < 1% was negative and ≥ 1% was positive. Ki67 < 20% was low expression and ≥20% was high expression (19). The staining site of HER2 was at the cell membrane, and staining of 0 or 1+ was defined as “HER2-negative” and 3+ was defined as “HER2-positive”. For specimens staining 2+ by IHC analyses, further confirmation was obtained by fluorescence in situ hybridization (FISH): “FISH non-amplification” was defined as HER2-negative and “FISH amplification” was defined as HER2-positive.

**Supplemental data 2**

**Scanning parameters.**

FS-T2WI parameters: repetition time (TR) = 4000 ms, echo time (TE) = 70 ms, matrix = 192 × 192, field of view (FOV) = 360 mm × 360 mm, layer thickness = 5.0 mm, layer spacing = 2.0 mm. DWI parameters: b = 800 s/mm^2^, TR =8300 ms, TE = 85 ms, matrix = 192 × 192, FOV = 360 mm × 360 mm, layer thickness = 5.0 mm, GAP = 2.0 mm. TIWI+C parameters: TR = 4.14 ms, TE = 1.42 ms, matrix = 320 × 192, FOV = 360 mm × 360 mm, layer thickness = 1 mm, layer spacing = 0.2 mm.

# Supplementary Figures and Tables

## Supplementary Figures


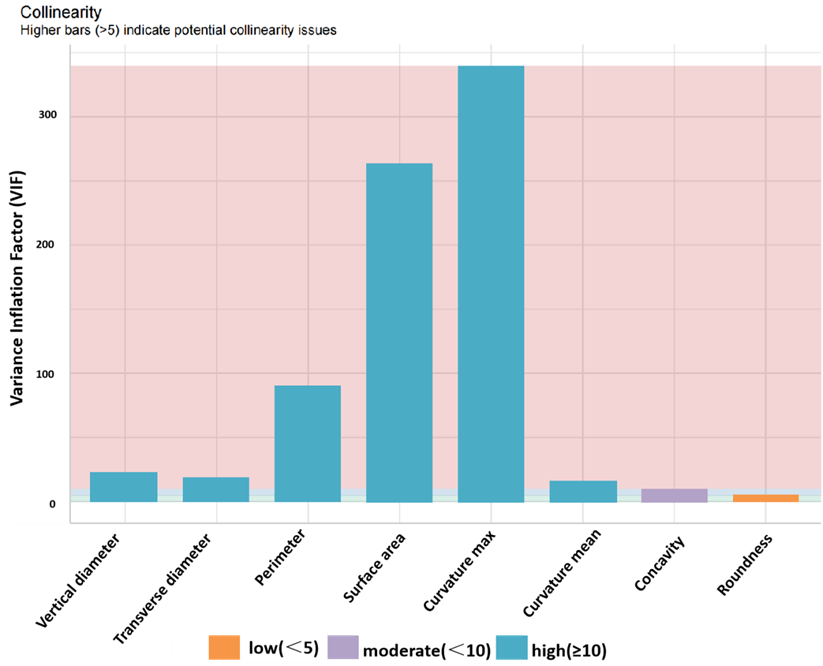


**FIGURE S1**| Multicollinearity analysis. Blue bars represent VIF greater than 10, purple bar represents VIF less than 10 and orange bar represents VIF less than 5. When VIF<10, there is no multicollinearity; when 10≤VIF<100, there is strong multicollinearity; when VIF≥100, there is severe multicollinearity. VIF, variance inflation factor.


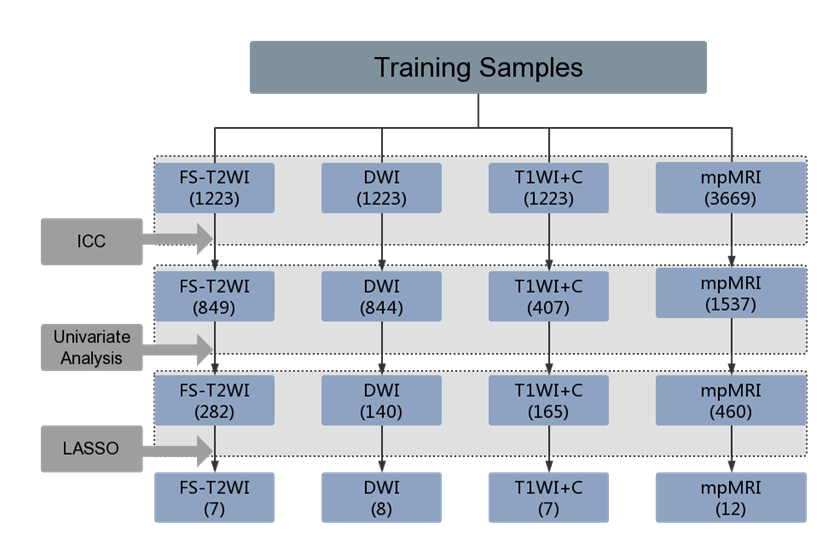


**FIGURE S2|** Flowchart for FS-T2WI, DWI, T1WI+C, and mpMRI showing reduction of feature dimensionality. The number of features remaining after dimensionality reduction is in parentheses.


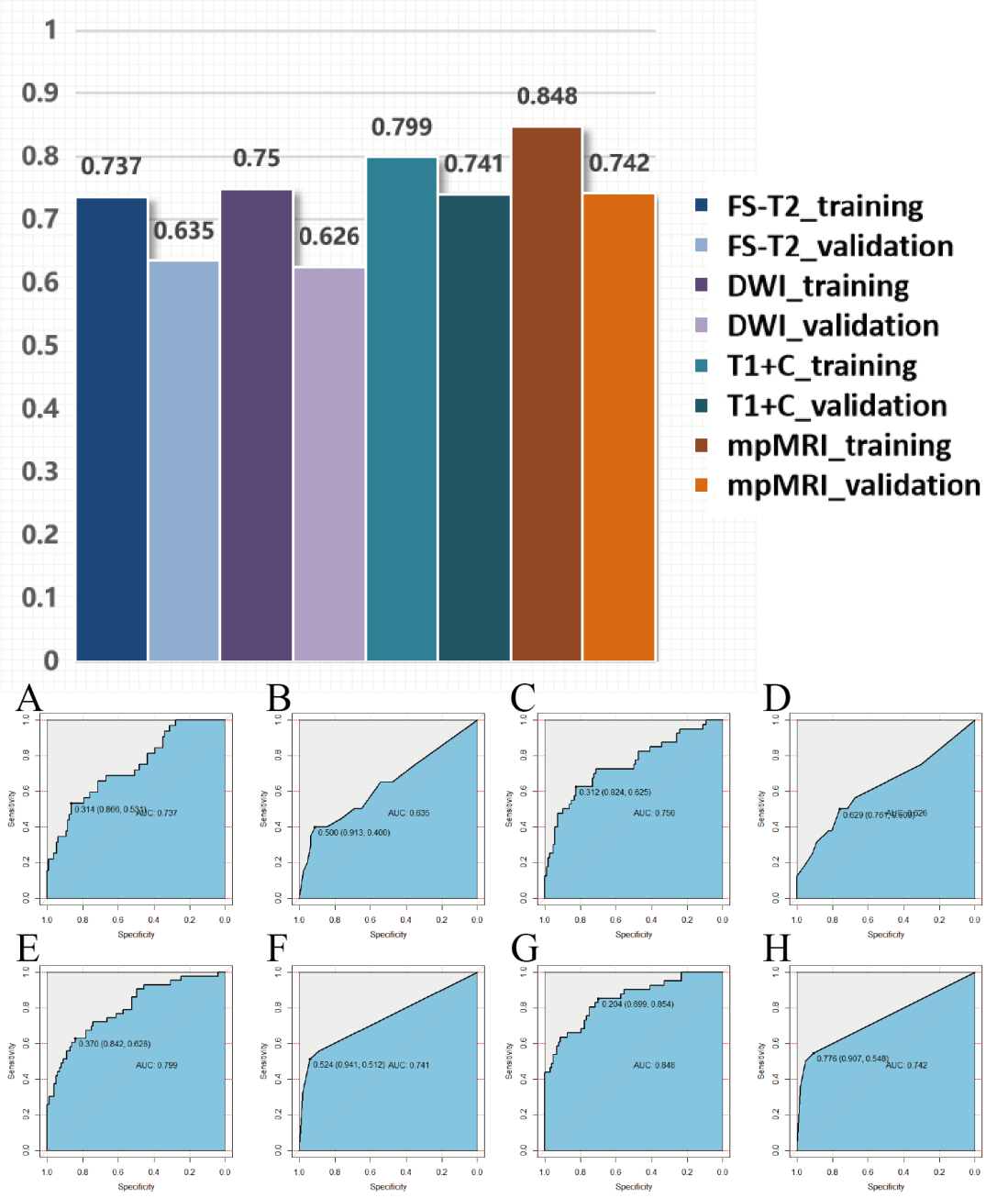


**FIGURE** **S****3|** ROC curves of radiomics signatures based on a single sequence and multiparametric MRI for pCR prediction in the training cohort and validation cohort. **(A)** training cohort of FS-T2WI. **(B)** validation cohort of FS-T2WI. **(C)** training cohort of DWI. **(D)** validation cohort of DWI. **(E)** training cohor of T1+C. **(F)** validation cohort of FS-T2WI. **(G)** training cohort of multiparametric MRI. **(H)** validation cohort of multiparametric MRI.

## 2.2 Supplementary Tables

**TABLE S****1** | Intra-group and inter-group agreement of quantitative parameters by two radiologists

| **Parameter** |  | **ICC (95%CI)** | ***P*-value** |
| --- | --- | --- | --- |
| Vertical diameter |  | 0.986 (0.976–0.991) | <0.001 |
| Transverse diameter |  | 0.987 (0.979–0.992) | <0.001 |
| Perimeter |  | 0.983 (0.973–0.990) | <0.001 |
| Surface area |  | 0.953 (0.923–0.971) | <0.001 |
| Roundness |  | 0.966 (0.946–0.981) | <0.001 |
| Concavity |  | 0.986 (0.977–0.991) | <0.001 |
| Curvature maximum |  | 0.957 (0.946–0.973) | <0.001 |
| Curvature mean |  | 0.964 (0.951–0.984) | <0.001 |

ICC, intraclass correlation coefficient;CI,confidence interval

**TABLE S2|** Naming scheme for each sequence for features

| **Selected feature and intercept** | **Modality** | **Coefficient** |
| --- | --- | --- |
| FS-T2WI sequence (7) |  |  |
| Intercept |  | −2.27688078 |
| Original/ngtdm/coarseness | 104 | 0.57748253 |
| Log-sigma-1-5-mm-3D/glcm/InverseVariance | 327 | −0.13826062 |
| Log-sigma-2-0-mm-3D/glrlm/GrayLevelNonUniformityNormalized | 444 | 0.85097760 |
| Wavelet-LHH/firstorder/Kurtosis | 671 | −0.29945033 |
| Wavelet-LHH/firstorder/Range | 677 | −0.05882062 |
| Wavelet-LHH/ngtdm/Coarseness | 775 | 0.25934663 |
| Wavelet-HLL/glszm/SmallAreaHighGrayLevelEmphasis | 842 | -0.04523489 |
| DWI sequence (8) |  |  |
| Intercept |  | −1.90694463 |
| Original/shape/maximum2DDiameterSlice | 7 | −0.44683391 |
| Original/gldm/dependenceVariance | 60 | −0.10057279 |
| Original/ngtdm/contrast | 106 | 0.82720857 |
| Log-sigma-2-0-mm-3D/firstorder/Kurtosis | 392 | 0.06430653 |
| Log-sigma-2-0-mm-3D/glszm/ZonePercentage | 473 | 1.25353890 |
| Wavelet-LLH/ngtdm/Coarseness | 569 | 0.59118382 |
| Wavelet-LHL/glszm/LargeAreaLowGrayLevelEmphasis | 651 | 0.36623874 |
| Wavelet-LLL/glszm/ZoneVariance | 1218 | 0.25958250 |
| T1WI+C sequence (7) |  |  |
| Intercept |  | 12.64870520 |
| Original/shape/Maximum2DDiameterSlice | 7 | −0.05269047 |
| Log-sigma-0-5-mm-3D/ngtdm/Strength | 200 | 0.18275346 |
| Log-sigma-1-0-mm-3D/glcm/Correlation | 224 | −2.03656589 |
| Log-sigma-0-5-mm-3D/glcm/Idn | 324 | −9.88690633 |
| Log-sigma-2-0-mm-3D/glcm/MCC | 424 | −2.06681321 |
| Log-sigma-2-0-mm-3D/glrlm/ShortRunLowGrayLevelEmphasis | 458 | 0.43295902 |
| Wavelet-LLH/gldm/DependenceVariance | 525 | −0.25299241 |
| Multiparametirc sequence (12) |  |  |
| Intercept |  | 4.59154672 |
| Original/shape/Maximum2DDiameterSlice | T1WI+C7 | −0.17457776 |
| Log-sigma-0-5-mm-3D/ngtdm/Strength | T1WI+C200 | 0.12370077 |
| Log-sigma-1-0-mm-3D/glcm/Correlation | T1WI+C224 | −1.59333848 |
| Log-sigma-0-5-mm-3D/glcm/Idn | T1WI+C324 | −3.24587525 |
| Log-sigma-2-0-mm-3D/glrlm/ShortRunLowGrayLevelEmphasis | T1WI+C458 | 0.94972916 |
| Wavelet-LHL/gldm/DependenceVariance | T1WI+C618 | −0.47987897 |
| Wavelet-HLL/gldm/DependenceVariance | T1WI+C804 | −0.53138627 |
| Original/gldm/DependenceVariance | DWI60 | −1.03259897 |
| Original/gtdm/Contrast | DWI106 | 0.3502368 |
| Wavelet-LHL/glszm/LargeAreaLowGrayLevelEmphasis | DWI651 | 0.10193912 |
| Log-sigma-1-5-mm-3D/ngtdm/Strength | FS-T2WI386 | 0.04867799 |
| Wavelet-LHL/ngtdm/Coarseness | FS-T2WI662 | 0.05095310 |
